# A Men's Workplace Health Intervention: Results of the POWERPLAY Program Pilot Study

**DOI:** 10.1097/JOM.0000000000000793

**Published:** 2016-08-08

**Authors:** Steven T. Johnson, Sean Stolp, Cherisse Seaton, Paul Sharp, Cristina M. Caperchione, Joan L. Bottorff, John L. Oliffe, Margaret Jones-Bricker, Sonia Lamont, Kerensa Medhurst, Sally Errey, Theresa Healy

**Affiliations:** Centre for Nursing and Health Studies, Athabasca University (Dr Johnson); Institute for Healthy Living and Chronic Disease Prevention (Mr Stolp, Dr Seaton, Mr Sharp, Dr Caperchione, Dr Bottorff), School of Health and Exercise Sciences, University of British Columbia, Kelowna (Dr Caperchione); School of Nursing, University of British Columbia, Vancouver (Dr Oliffe); Canadian Cancer Society, BC and Yukon Division, Prince George (Ms Jones-Bricker); Prevention Programs (Ms Lamont, Ms Errey), and Office of Research, British Columbia Cancer Agency (Ms Medhurst); Population Health Department, Northern Health, Prince George (Dr Healy), Canada; and Faculty of Health Sciences, Australian Catholic University, Melbourne, Australia (Dr Bottorff).

## Abstract

**Objective:**

To explore physical activity and eating behaviors among men following the implementation of a gender-sensitive, workplace health promotion program.

**Methods:**

Using a pre-post within-subjects design, computer-assisted telephone interviewing (CATI) was used to collect health-related information along with physical activity and fruit/vegetable intake at baseline and after 6 months.

**Results:**

At baseline, participants (*N* = 139) consumed 3.58 servings of fruit and vegetables/day and engaged in an average of 229.77 min/week moderate-vigorous physical activity (MVPA). At 6 months, daily fruit/vegetable intake did not increase, whereas MVPA increased by 112.3 min/week.

**Conclusions:**

The POWERPLAY program successfully increased weekly MVPA. Engaging men in health promotion can be a challenge; here, the workplace served as a valuable environment for achieving positive change.

Regular physical activity and healthy eating are well known to support the prevention of many chronic diseases including type 2 diabetes, cardiovascular disease, and cancer.^1,2^ Unfortunately, achieving a lifestyle that follows recommended guidelines for physical activity and healthy eating can be elusive for many men, especially those in rural and northern regions.^3^ Males in northern British Columbia (BC), Canada are at particularly high risk for chronic disease because of lifestyle factors. In this region, in 2011/2012, 68% of men were overweight or obese, substantially higher than the proportion of women (51%).^4^ Compared with 49% of their female counterparts, only 26% of northern BC men consumed five or more servings of fruit and vegetables per day in 2014.^4^ Furthermore, slightly over half of men (53%) in the same region achieve sufficient physical activity for health benefit.^4^ Compounding the risk for chronic disease, men residing in rural and northern regions are also considered “hard to reach” when considering health promotion strategies geared to support the achievement of public health guidelines for disease prevention.

Although lifestyle interventions have been developed and evaluated in randomized controlled trials, men represent a low proportion of participants (27%) in these studies.^5^ This lack of success in engaging men in lifestyle behavior changes has been linked to the failure to account for gender in the design and delivery of health promotion initiatives.^6–8^ Accordingly, efforts are now being directed to develop and evaluate approaches to promote health behavior change that are specifically designed to overcome the disengagement that men have displayed for general health promotion strategies.^9^ For example, health promotion interventions targeting men have recently shown an increase in the use of gender-sensitive approaches and novel engagement strategies that incorporate men's preferences and interests.^7^ Emerging evidence also suggests that men are more likely to participate in health promotion programs in settings they are familiar with (eg, worksite or sports clubs) and in programs that provide support and deliver interventions in partnership with trusted groups.^9^

Based on a review of the literature,^7^ consultations with stakeholders, and feedback obtained through focus groups held with men in northern BC,^10^ we developed and implemented a novel gender-sensitive program (called POWERPLAY) aimed at increasing daily physical activity and fruit and vegetable intake among men in male-dominated worksites.^11^ POWERPLAY was specifically designed to appeal to rural men in northern BC. The purpose of this pilot study was to explore physical activity and healthy eating behaviors among men following the implementation of an evidence and focus group informed gender-sensitive, workplace health promotion program across four male-dominated worksites in northern British Columbia, Canada. We hypothesized that at 6 months, weekly moderate-vigorous physical activity (MVPA), and daily fruit and vegetable intake would increase after implementing the POWERPLAY program.

## METHODS

### Study Design

Detailed design and rationale for the study have been previously reported.^11^ Briefly, a quasi-experimental pre-post within-subjects design was used to evaluate the POWERPLAY workplace health promotion program. The study protocol was approved by the University of British Columbia Behaviour Research Ethics Board (#H13–02408) and the Northern Health Research Review Committee (RRC-2014–0015).

Worksites in northern BC were invited to participate based on proportion of male employees (ie, >50%), and their existing relationships with community partners and research team members. Four worksites in northern BC offered the POWERPLAY program and participated in this study. These included a regional municipality, two trucking companies, and a shipping terminal. After employers agreed to offer the POWERPLAY program, male employees 18 years of age or older were recruited. For recruitment, posters with gender-sensitive messaging framed in part with input from focus groups held with northern BC men^10^ were used to raise workplace awareness about the launch of the POWERPLAY program. Prior to the start of the program, a launch event was held at each worksite and included confidential health screenings (ie, blood pressure and heart rate) provided by nurses which served to introduce and gain momentum for the program. Program sign-up sheets were left after the launch events for any additional men to consent to being contacted. Rolling recruitment occurred between September 2014 and October 2014. Men who provided contact information were contacted by telephone, provided further information about the study, and invited to complete the telephone survey. The study commenced in October 2014 and follow-up data collection was completed in April 2015 (six months after the start of the POWERPLAY program, and 2 months after the end of the second 6-week challenge) via computer-assisted telephone interviewing (CATI) surveys. Following completion of each survey, participants were mailed a gift card ($20), and their names were entered into a draw for a vacation (value of $1000). The POWERPLAY program was offered in all of the worksites; there was no randomization and the study was not blinded.

### Intervention

The POWERPLAY program encouraged a progressive increase in daily physical activity and healthy eating (eg, focusing mainly on increasing fruit and vegetable intake). To support these behavior changes, worksites were provided with print-based materials and implementation resources (eg, weekly Toolbox Tips, tracking posters, team logbooks). The program was implemented over a 6-month period and included two, 6 weeks challenges during that time. Both challenges included friendly competition between employees and also between worksites as well as tools (eg, resources for tracking progress, pedometers) to assist with self-monitoring of physical activity and healthy eating behaviors. All resources and materials were designed to be gender-sensitive, incorporating a masculine and a northern BC look and feel and providing clear messaging around physical activity and healthy eating.

### Measures

#### Socio-Demographic Characteristics

A series of questions were used to describe the sample including age, marital status, ethnicity, education, income, occupation, history of health conditions, and smoking status. Participants were also asked to report their current height and weight, from which a body mass index was calculated in kg/m^2^. The Occupational Sitting and Physical Activity Questionnaire^12^ was used to assess the proportion of time during the work day spent sitting, standing, walking, and doing physically demanding tasks.

#### Physical Activity

To assess physical activity the leisure score index (LSI) of the Godin Leisure-Time Exercise Questionnaire^13^ was included. The LSI contains three questions that assess both the average frequency and duration of mild, moderate, and strenuous (vigorous) activities during free time over a typical week during the past month. The LSI demonstrated a 1-month test-retest reliability of 0.62 and concurrent validity coefficients of 0.32 with an objective indicator (CALTRAC accelerometer), 0.56 with VO_2max_ (as measured by expired gases), and −0.43 with percent body fat (as measured by hydrostatic weighing). An independent evaluation of this measure found its degree of reliability and validity compared favorably with nine other self-report measures of physical activity based on various indices.^14^

#### Healthy Eating Behavior

Participants were asked to report on the number of daily servings of fruits and vegetables they usually consume in a day.^15^

#### Self-Efficacy

Regular physical activity self-efficacy was assessed by asking participants “over the next 6 months, how confident are you that you can participate in regular physical activity on no less than 5 days of the week?”^16^ Healthy eating self-efficacy was assessed through two questions adapted from a previous measure.^16^ One asked participants how confident they were in eating two servings of fruit a day and the other asked participants how confident they were in eating five servings of vegetables a day. All three questions were rated on a Likert scale ranging from 1 (not at all confident) to 5 (extremely confident).

### Analysis

Using last-observation carried forward, repeated measures ANOVAs were used to compare baseline data and 6 months data on all measures. Extreme cases (*n* = 1 to 3) for the outcome measures were removed from the analyses.^17^ Chi-square test was used to compare proportional differences for categorical data (ie, meeting physical activity guidelines vs not). A *P*-value of less than 0.05 was considered statistically significant.

## RESULTS

**FIGURE 1:**
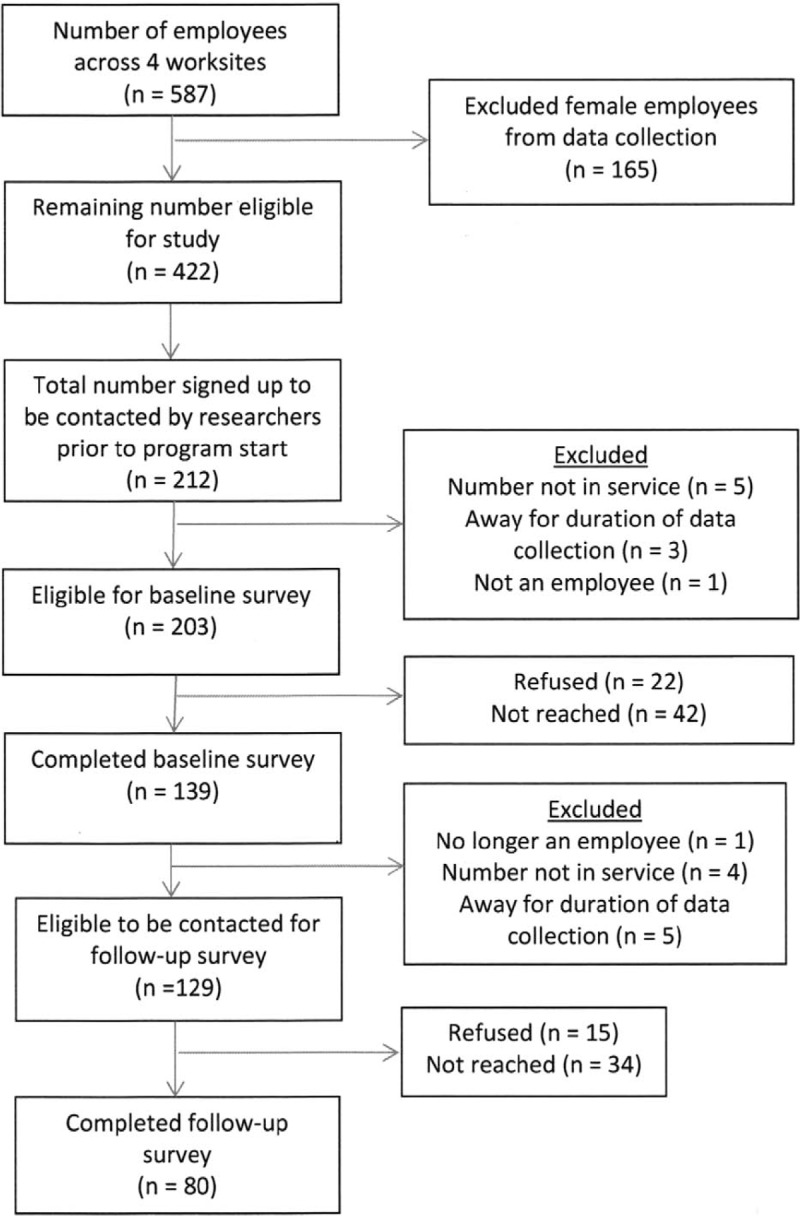
Participant flow chart.

Prior to the POWERPLAY program implementation, 203 eligible participants agreed to be contacted by researchers. At baseline, 139 completed surveys (response rate 68%). The proportional distribution was relatively equal across the four worksites (*N* = 29, 31, 39, and 40). At 6 months, 80 participants completed follow-up surveys for a loss to follow-up of 42%. See Fig. 1 for a diagram of participant recruitment. At baseline, participants (*N* = 139) ranged in age from 18 to 66 (*M* = 43.71; *SD* = 12.51); had a body mass index 28.70 (*SD* = 4.37) kg/m^2^; consumed 3.58 (*SD* = 2.02) servings of fruit and vegetables/day; and, engaged in 229.77 minutes of weekly MVPA. At baseline, the number of participants who reported being informed by a health care provider that they had (or previously had) a health condition, included 8 with Type 2 Diabetes, 31 with high cholesterol, 36 with high blood pressure, 13 with digestive problems, 11 with a mental health or mood disorder, 7 with heart problems, 18 with arthritis, 55 with knee/hip/back problems, 3 diagnosed with cancer, and 11 with other health problems. No participant reported having had a stroke and 20 men were smokers (16 regular smokers and 4 occasional smokers). See Table 1, for a full breakdown of participant demographic characteristics.

### Physical Activity

Participation in weekly vigorous physical activity based on the Godin Leisure-Time Exercise Questionnaire increased by 58.98 minutes per week, *F* (1, 137) = 20.65, *P* < 0.001, *ή*^2^ = 0.13 and moderate physical activity increased by 53.30 minutes per week, *F* (1, 137) = 24.92, *P* < 0.001, *ή*^2^ = 0.15. See Table 2 for means and confidence intervals. After 6 months, 72% of men were meeting recommended levels of moderate to vigorous physical activity (ie, 150 min/week) compared with 58% at baseline, *χ^2^* (1, 137) = 11.86, *P* = 0.001.

### Healthy Eating Behavior

No significant differences between time points were found for the number of servings of fruits and vegetables consumed in a usual day (*P* = 0.23). Means and confidence intervals can be seen in Table 2.

### Self-Efficacy

As shown in Table 2, self-efficacy for physical activity increased (*F* [1, 138] = 4.04, *P* = 0.046, *ή*^2^ = 0.03) but self-efficacy for fruit (*P* = 0.76) and vegetable (*P* = 0.41) intake did not change.

## DISCUSSION

The purpose of this pilot study was to explore physical activity and healthy eating behaviors among men following the implementation of an evidence and focus group informed gender-sensitive, workplace health promotion program across four male-dominated worksites in northern British Columbia, Canada. We hypothesized that at 6 months, weekly minutes of MVPA, and daily fruit and vegetable intake would increase after implementing the POWERPLAY program. Our pilot study revealed an increase in weekly MVPA but no change in fruit and vegetable intakes at the 6 months follow-up.

The study results align with the current literature around promoting physical activity among men. For example, we used promotional and educational materials, self-monitoring and challenges (ie, a 6-week pedometer-based challenge) to increase MVPA in the POWERPLAY program.^11^ The existing literature^7^ and our focus group consultations with men^10^ suggested these approaches would be successful and indeed they were; that is, tailoring the program not only to the specific men, but also to the specific location where these men work and live, making it more accessible and of interest to them.^18,19^ Here, we further assert that the promotion of physical activity using the POWERPLAY program was successful since it considered men's interests and preferences while considering masculine ideals and gender influences. The workplaces that offered the POWERPLAY program provided incentives (eg, prizes) for participation, and research has revealed that offering incentives for workplace wellness programs focused on prevention is effective.^20^ Moreover, physical activity was aligned with work performance and productivity, and in this regard increasing physical activity was strength based, offering to extend an array of familiar benefits to the men. The fact that increases in physical activity were evident 2 months following completion of the last 6-week challenge indicates that the program promoted sustained behavior change. The increase in physical activity is also notable given the study occurred over the winter months in northern locations that typically experience heavy snow falls. We suggest other researchers should consider this type of approach when promoting physical activity among men.

Increasing fruit and vegetable intake for men is challenging. In their systematic review, Taylor et al^8^ suggested better outcomes could be achieved when the provision of hard numbers or quantitative information on dietary elements are provided to men; as well, they suggest the inclusion of self-monitoring and tailored feedback to be beneficial for improving elements of the diet among men. Others have also suggested very specific food-based guidelines are required for men; that is, males respond to very direct statements such as—“Eat this or don’t eat this” or “Drink less”.^21^ Further, some suggest humor is an important strategy for promoting healthy eating among men, and this was exemplified in a successful program called Preventing Obesity Without Eating like a Rabbit where men indicated they did not actually have to “eat like rabbits” to lose weight.^22^ Understanding why men did not increase their fruit and vegetable intake in the current study is curious. One possibility is that we did not incorporate sufficient self-monitoring for fruit and vegetable intake like we did for increasing MVPA (ie, my playbook, pedometers, and workplace tracking posters). In addition, efforts to change workplace policies related to the availability and promotion of fruit and vegetables in cafeterias and vending machines may also be needed to complement other approaches. Additionally, it has been suggested that young males report physical health, appearance, and social influences as important motivators for eating healthy,^23^ while factors such as cost, peer influence, and extra effort can pose as significant barriers. Recent research has revealed a link between fruit and vegetable consumption and lower erectile dysfunction,^24^ lower depression,^25^ and lower hypertension.^26^ Introducing men to some of the less well-known social and emotional benefits of eating more fruits and vegetables holds potential to motivate increased consumption, but more research is needed on this topic. Men in the current study reported not being confident (eg self-efficacy) in increasing their fruit and vegetable intake. It is plausible that cost and availability (eg, high cost in northern communities) influenced their confidence since focus group participants expressed these as being salient barriers when trying to eat healthy.^10^ Although a small but growing trend in offering workplace health promotion programs targeting healthy eating and active living among men has been noted in the literature, there remains uncertainty about the most effective nutrition interventions for males.^8^ Future research must tease out how best to promote healthy eating among men.

The current study has many strengths. First, POWERPLAY was informed by the current literature and by consultation with men likely to be program users.^10^ Second, the program was implemented in the “real world”; that is, four male-dominated worksites agreed to adopt and implement POWERPLAY and the program was strictly voluntary. Third, both behavioral and informational health promotion strategies were employed. Despite these identified strengths, we acknowledge this was not a randomized controlled trial. In addition, the intervention was voluntary for men in these worksites and hence participant exposure may have varied across the worksites. Variations in exposure may also explain the loss to follow-up in that men who experienced limited exposure to the program may have been less interested in completing the follow-up survey. Nevertheless, the results suggest the POWERPLAY program when deployed in worksites can increase weekly MVPA among men. We believe our results have external validity but perhaps only to worksites that are located in rural and/or northern locations and male-dominated. Our sample of participants was also primarily well-off financially. Hence, POWERPLAY may not be applicable to urban male-dominated worksites or those employed in lower-paying jobs; however further research is required in this regard.

## CONCLUSIONS

In summary, the geographic location and the workplace served as a valuable environment for achieving positive change in physical activity among men. The gender-sensitive approach used in POWERPLAY holds great potential for extending health promotion programs to a range of male-dominated workplaces and may have application to other types of underserved men's groups.

## Figures and Tables

**TABLE 1:** Participant Characteristics for the POWERPLAY Program (*N* = 139)

	Mean (SD)/%
Age	43.71 (12.5)
Marital status	
Married/Common-Law	71.1%
Single	23.0%
Separated/Widowed	5.7%
Ethnicity	
Caucasian	83.5%
First Nation/Metis/Inuit	8.6%
Other	7.9%
Education	
Post-secondary certificate or diploma	52.2%
High school or less	44.9%
Graduate degree	2.9%
Income	
>$80,000	66.9%
<$80,000	27.3%
No response	5.8%
Occupation	
Truck drivers	31.7%
Tradespersons (eg, mechanics)	29.5%
Heavy equipment operators	12.2%
Mangers	7.9%
Firefighters/fire chiefs	6.5%
Other (eg, lifeguard, laborer, etc)	12.2%
Proportion of workday spent:	
Sitting	53.6%
Standing	16.6%
Walking	18.8%
Heavy labor	11.1%

SD, standard deviation.

**TABLE 2:** Six-Month Follow-Up for the POWERPLAY Program

	Baseline Mean [CI]	Six-Month Mean [CI]	*F*
Weekly physical activity (min)			
Vigorous	119.82 [89.00, 150.64]	178.80 [142.38, 215.23]	20.65^**^
Moderate	109.95 [89.77, 136.76]	163.25 [136.76, 189.74]	24.92^**^
Mild	192.95 [156.68, 229.22]	191.73 [156.71, 226.75]	0.004
Self-efficacy physical activity	4.07 [3.91, 4.24]	4.20 [4.05, 4.35]	4.04^*^
Daily fruit and vegetable servings	3.58 [3.22, 3.94]	3.40 [3.07, 3.74]	1.45
Self-efficacy for two servings of fruit/day	4.21 [4.06, 4.35]	4.23 [4.10, 4.36]	0.10
Self-efficacy for five servings of vegetables/day	3.24 [3.04, 3.43]	3.30 [3.11, 3.49]	0.68

Repeated Measures ANOVAs with baseline observations carried forward were conducted (*N* = 136 to 139 depending on number of outliers removed). CI, confidence interval.

^*^P < 0.05.

^**^P < 0.001.
